# Pseudoprogression in Hepatocellular Carcinoma During Atezolizumab Plus Bevacizumab Therapy: A Case Report and a Review of Literature

**DOI:** 10.7759/cureus.86905

**Published:** 2025-06-28

**Authors:** Mikee Elaine U Wee, Chao-Hung Hung, Ming-Chao Tsai, Chien-Hung Chen, Yuan-Hung Kuo

**Affiliations:** 1 Department of Internal Medicine, Section of Gastroenterology and Digestive Endoscopy, Manila Doctors Hospital, Manila, PHL; 2 Division of Hepatogastroenterology, Department of Internal Medicine, Kaohsiung Chang Gung Memorial Hospital and Chang Gung University College of Medicine, Kaohsiung, TWN

**Keywords:** atezolizumab plus bevacizumab, case report, hepatocellular carcinoma, immune checkpoint inhibitors, pseudoprogression

## Abstract

Pseudoprogression is an atypical response pattern to immune checkpoint inhibitors (ICIs), characterized by initial tumor enlargement or the appearance of new lesions, followed by subsequent tumor regression. While this phenomenon has been observed in several solid tumors, its occurrence in hepatocellular carcinoma (HCC) is rare and not well understood, presenting diagnostic and therapeutic challenges.

We report the first female case of HCC pseudoprogression during atezolizumab and bevacizumab therapy. The patient is a 63-year-old woman with chronic hepatitis B-related advanced HCC. After multiple prior treatments, she was started on combination therapy with atezolizumab and bevacizumab. Following four treatment cycles, imaging revealed tumor enlargement and new small lesions, suggestive of disease progression. However, her serum alpha-fetoprotein (AFP) level had decreased from 45,598 ng/mL to 23,719 ng/mL, and there was no clinical deterioration. Based on these findings, treatment was continued. Imaging after eight and 12 cycles demonstrated marked tumor regression and the normalization of AFP (<2 ng/mL), confirming a diagnosis of pseudoprogression.

Although uncommon, pseudoprogression should be considered during ICI therapy for HCC. The accurate interpretation of radiologic findings in conjunction with clinical status and tumor markers is essential to avoid the premature discontinuation of potentially effective treatments. Further research is warranted to elucidate the underlying mechanisms, predictive markers, and clinical significance of pseudoprogression in HCC.

## Introduction

Pseudoprogression is an atypical tumor response characterized by an initial increase in tumor size or the emergence of new lesions, followed by subsequent regression [1]. This phenomenon is hypothesized to result from T-cells infiltrating into the tumor, leading to temporary lesion enlargement as part of an immune-mediated therapeutic response [2,3]. Unfortunately, this can be misinterpreted as treatment failure, potentially resulting in the premature discontinuation of effective therapies.

With the growing use of immune checkpoint inhibitors (ICIs) in cancer treatment, recent research has focused on better identifying pseudoprogression, leading to the development of immune-related response evaluation criteria (irRECIST) to differentiate it from true tumor progression [1]. However, a retrospective multicenter study suggests that continuing nivolumab treatment in advanced hepatocellular carcinoma (HCC) patients with unconfirmed progressive disease may offer limited clinical benefit due to the low likelihood of pseudoprogression [4]. Currently, for patients with advanced or unresectable HCC, combination therapy with the anti-programmed cell death-ligand 1 (anti-PD-L1) agent atezolizumab and the anti-vascular endothelial growth factor (anti-VEGF) A agent bevacizumab has shown superior progression-free and overall survival compared to sorafenib and is now considered the standard systemic therapy in this setting [5]. Although rare in HCC compared to other solid tumors, pseudoprogression associated with ICIs, particularly atezolizumab plus bevacizumab, remains poorly understood and presents a diagnostic and therapeutic challenge.

Herein, we present a compelling case of pseudoprogression in a patient with HCC treated with atezolizumab plus bevacizumab, followed by a remarkable therapeutic response. We also provide a literature review to enhance the understanding of previously reported cases. This case illustrates the diagnostic complexity of pseudoprogression in HCC during atezolizumab plus bevacizumab therapy, particularly in a female patient, a demographic rarely represented in existing reports. It highlights the limitations of conventional imaging-based response criteria and underscores the importance of integrating clinical status and tumor markers into treatment evaluation.

## Case presentation

A 63-year-old Taiwanese woman was referred to our service for continuity of care. She was a case of HCC stage T1N0M0 and Barcelona clinic liver cancer (BCLC) stage A on the background of hepatitis B on tenofovir alafenamide, who underwent lateral segmentectomy and cholecystectomy. Postoperatively, she had experienced multiple episodes of tumor recurrence with tumor thrombus and underwent radiofrequency ablation and radiotherapy on separate episodes, despite starting on lenvatinib and having it discontinued later on due to tumor progression.

**Figure 1:**
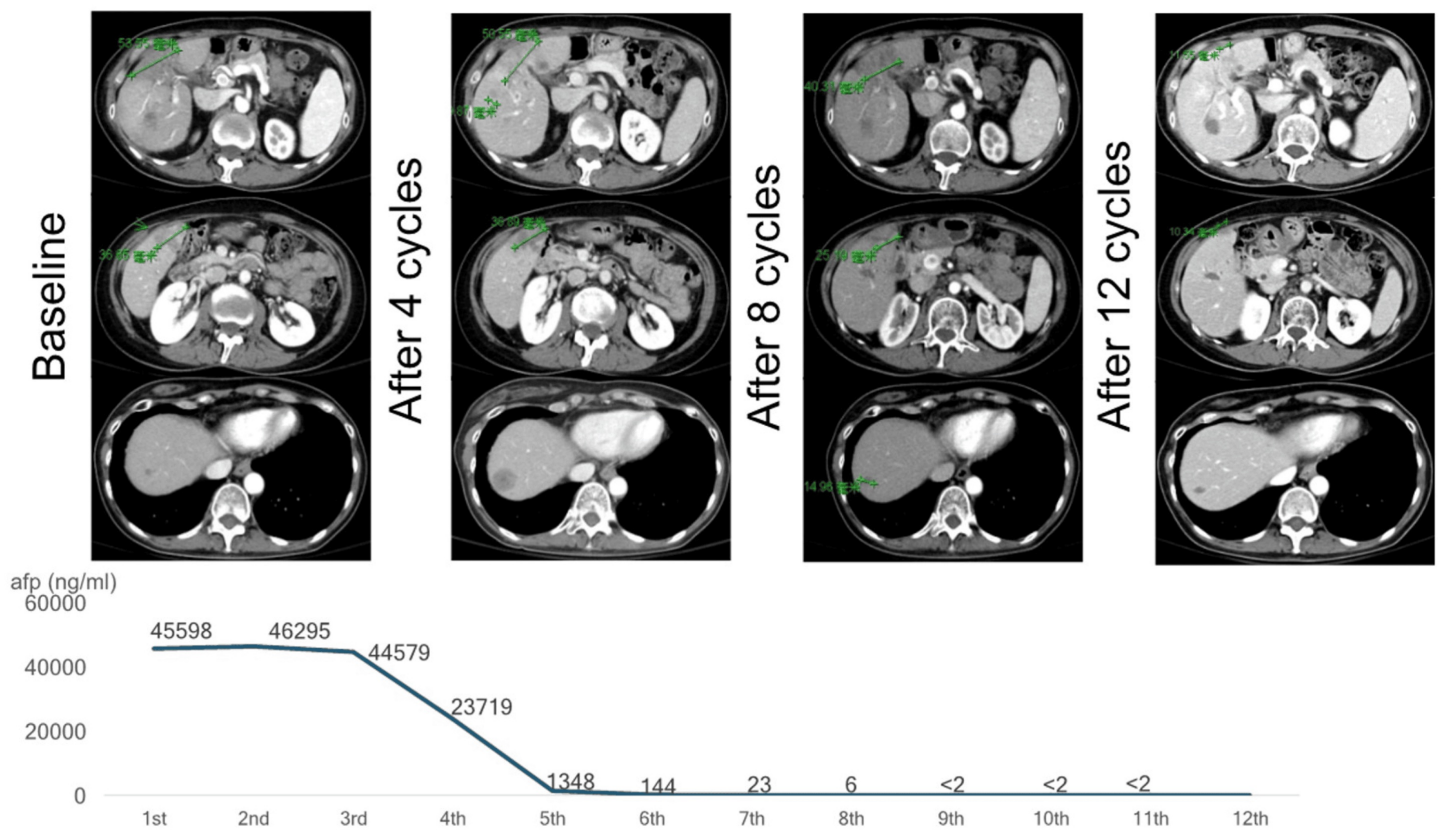
Patient serum AFP trends and CT imaging while on atezolizumab and bevacizumab AFP: alpha-fetoprotein

Four years post operation, she was again diagnosed with three HCC tumor recurrences seen on CT scan (unidimensional at 5.4 cm, 3.7 cm, and <1.0 cm at segments 4-5, segment 5, and segment 7, respectively), with concurrent serum alpha-fetoprotein (AFP) level of 45,598 ng/mL. The patient was diagnosed with a case of advanced, unresectable HCC. She was advised to undergo immunotherapy (atezolizumab 1,200 mg and bevacizumab 500 mg). After four cycles of immunotherapy, there was a significant increase in the size of one of the tumors (unidimensional from <1.0 cm to ~3.0 cm at segment 7), despite a contradicting significantly decreased AFP level at 23,719 ng/mL. The patient was still advised to continue her current treatment. After eight cycles of immunotherapy, there were a modest decrease in the size of the tumors (unidimensional at 4.0 cm, 2.5 cm, and 1.5 cm) and a drastic decrease in the serum AFP level to 6 ng/mL. As her immunotherapy reached the 12th cycle, there was a notable partial response: progressive decrease in both the size of the tumors of 78%, 73%, and 30% (unidimensional at 1.2 cm, 1.0 cm, and 1.3 cm, respectively) and serum AFP level to <2 ng/mL. By RECIST 1.1 or irRECIST criteria, the patient has exhibited a partial response or an immune-related partial response, respectively. To date, the patient has been continuously receiving atezolizumab and bevacizumab without any minor or major adverse events with close monitoring and follow-up. She has been doing well and was able to perform her usual daily activities (Figure 1).

## Discussion

Pseudoprogression is an atypical response pattern observed in various malignancies, including melanoma (6.4%), non-small cell lung cancer (5%), and genitourinary cancers (7%) among patients receiving ICI therapy [6]. Although pseudoprogression is rarely reported in HCC, the awareness of this phenomenon is increasing as the use of ICIs becomes more widespread. In this report, we describe a 63-year-old woman with hepatitis B-related, advanced, unresectable HCC who was initially treated with lenvatinib as first-line therapy. Following the fourth cycle of atezolizumab plus bevacizumab, imaging suggested disease progression, despite a concurrent decline in serum AFP levels. However, subsequent imaging after the eighth and 12th cycles revealed a marked reduction in both tumor burden and AFP levels, confirming pseudoprogression. This case underscores the importance of cautiously interpreting early imaging results and highlights the need for prolonged monitoring before modifying treatment plans.

A comprehensive literature review using the PubMed database identified seven published case reports of HCC pseudoprogression associated with atezolizumab plus bevacizumab therapy [3,7-12]. These cases are summarized in Table 1. Most patients were older adults, except one case involving a younger individual with fibrolamellar HCC [8]. Notably, our case is the only reported instance involving a female patient. Interestingly, the majority (62.5%) of pseudoprogression cases occurred in patients without underlying hepatitis B or C infection. Only three cases involved viral hepatitis: two with hepatitis C and our current case with hepatitis B [9,10].

**Table 1:** Summary of reported cases of HCC pseudoprogression on atezolizumab plus bevacizumab therapy PsP, pseudoprogression; AFP, alpha-fetoprotein; LN, lymph node; PV, portal vein; PR, partial response; CR, complete response; HCC, hepatocellular carcinoma; BCLC, Barcelona clinic liver cancer; RHV, right hepatic vein; SVR, sustained virological response; TACE, transarterial chemoembolization; RT, radiation therapy

Year	Reference	Profile	Case	Etiology	Prior treatment	Baseline AFP level (ng/mL)	Time of PsP	Type of PsP	AFP level (ng/mL) at the time of PsP	Response (RECIST 1.1)
2021	[7]	74/male	HCC with lung, bilateral iliac, and pelvic LN metastasis	Alcoholic	TACE	~4,000	After the second course	Increased size of the lung, bilateral iliac, and pelvic LN metastatic tumors; increased number of lung metastases	~6,500	PR
2022	[8]	30/male	Fibrolamellar HCC with LN metastasis	Non-viral	None	9.8	After the fourth course	Increased size of the LN metastasis	Normalization	PR
2023	[9]	56/male	BCLC stage B HCC	Alcoholic liver and hepatitis C (SVR)	TACE	~20	After the third course	Increased size of tumors	~80	CR
2023	[10]	70/male	HCC with lung metastasis, PV invasion, and RHV invasion	Hepatitis C	TACE, ×2	8,416	After the second course	Increased size of tumor; progression of vascular invasion; increased number of lung metastases	~8,500	PR
2024	[11]	70/male	Moderately differentiated HCC with microvascular invasion	Non-viral	Hepatectomy	39.1	After the second course	Increased size of tumors	6	PR
2024	[12]	62/male	HCC with lung metastasis	Non-viral	Hepatectomy	~9,000	After the fourth course	Increased size of liver tumor; increased size of lung metastasis	Decreased	CR (lung) and PR (liver)
2025	[3]	65/male	Poorly differentiated HCC with lung metastasis, with cervical and abdominal LN metastasis	Alcoholic	None	37,671	After four days	New liver tumors	Not tested	PR
Current case		63/female	HCC with PV thrombus	Hepatitis B	Hepatectomy, RT, and lenvatinib	45,598	After the fourth course	Increased size of one tumor and new lesions	23,719	PR

The pathogenesis of HCC pseudoprogression remains poorly understood, and its association with underlying etiology is an area of active inquiry. Some evidence suggests that the etiology of HCC may influence the response to ICIs, with potentially reduced efficacy observed in non-viral HCC, particularly those associated with nonalcoholic fatty liver disease [13]. However, current randomized controlled trials have not been stratified by etiology, and such findings derive from post hoc, non-prespecified subgroup analyses [5]. Whether non-viral etiologies are more prone to pseudoprogression remains an open question warranting further investigation.

While studies in other malignancies suggest that tumor markers can help support a diagnosis of pseudoprogression, the utility of AFP and protein induced by vitamin K absence-II (PIVKA-II) in HCC is not yet well established. In our review, although half of the reported cases demonstrated a declining AFP level during pseudoprogression, two cases exhibited increased AFP, and one showed stable levels. These findings suggest that tumor markers alone may not reliably distinguish pseudoprogression from true progression. Nonetheless, clinical improvement alongside declining AFP levels, despite radiologic progression, should raise suspicion for pseudoprogression [3,12]. As a single case report, the findings are inherently limited in generalizability by the absence of biopsy confirmation and long-term follow-up. Emerging techniques such as radiomics and the identification of predictive biomarkers, including immune-related gene signatures or circulating tumor DNA, may offer valuable tools to differentiate pseudoprogression from true progression in HCC [14,15]. Although still in early stages of validation, these approaches warrant further investigation to enhance clinical decision-making during immunotherapy.

## Conclusions

To our knowledge, this is the first reported case involving a female patient with hepatitis B-related HCC showing pseudoprogression undergoing treatment with atezolizumab plus bevacizumab. Differentiating pseudoprogression from true disease progression in HCC patients receiving immunotherapy remains a major clinical challenge. Recognizing this phenomenon is critical to prevent the premature discontinuation of potentially effective treatments. Ongoing research and the accumulation of clinical data are essential to enhance our understanding of the incidence, pathogenesis, and clinical relevance of pseudoprogression, as well as to establish optimal diagnostic and management strategies in HCC.
